# Design and Development of Enhanced Antimicrobial Breathable Biodegradable Polymeric Films for Food Packaging Applications

**DOI:** 10.3390/polym13203527

**Published:** 2021-10-14

**Authors:** Mona M. Abd Al-Ghani, Rasha A. Azzam, Tarek M. Madkour

**Affiliations:** 1 Department of Chemistry, School of Science and Engineering, The American University in Cairo, Cairo 11835, Egypt; monamagdi80@aucegypt.edu; 2 Department of Chemistry, Helwan University, Cairo 11795, Egypt; rasha_azzam@science.helwan.edu.eg

**Keywords:** micro-perforated food packaging, antimicrobial food packaging, sodium chloride nanocrystals, cinnamaldehyde, biodegradable polymers

## Abstract

The principle of breathable food packaging is to provide the optimal number of pores to transfer a sufficient amount of fresh air into the packaging headspace. In this work, antimicrobial microporous eco-friendly polymeric membranes were developed for food packaging. Polylactic acid (PLA) and polycaprolactone (PCL) were chosen as the main packaging polymers for their biodegradability. To develop the microporous films, sodium chloride (NaCl) and polyethylene oxide (PEO) were used as porogenic agents and the membranes were prepared using solvent-casting techniques. The results showed that films with of 50% NaCl and 10% PEO by mass achieved the highest air permeability and oxygen transmission rate (O_2_TR) with PLA. Meanwhile, blends of 20% PLA and 80% PCL by mass showed the highest air permeability and O_2_TR at 100% NaCl composition. The microporous membranes were also coated with cinnamaldehyde, a natural antimicrobial ingredient, to avoid the transportation of pathogens through the membranes into the packaged foods. In vitro analysis showed that the biodegradable membranes were not only environmentally friendly but also allowed for maximum food protection through the transportation of sterile fresh air, making them ideal for food packaging applications.

## 1. Introduction

Respiration and transpiration are the most important biochemical processes for fresh fruits and vegetables that strongly affect the shelf-life of fresh food. Atmospheric gas compositions within the packaging headspace play a significant role in fresh food preservation [1,2]. Therefore, modifying the composition of atmospheric gases within the packaging headspace is obtained through manipulating and adjusting the permeability of packaging material [3]. In general, adjustment of the permeability characteristics of fresh products packaging is important and can be achieved through two techniques, namely micro-perforated modified atmospheric packaging (MAP) and ventilated packaging. Each technique has its unique properties and applications in fresh food preservation.

Micro-perforated MAP is one of the MAP techniques that has gained considerable attention due to its low cost and high performance as a food preservation method. In this technique, packaging materials are provided with micro or macro perforates in order to regulate the gas transmission rates (GTRs). In fact, it was proven that micro-perforated films are able to achieve equilibrium concentrations of oxygen (O_2_) and carbon dioxide (CO_2_) gases inside the food packaging, which strongly affect several quality aspects of fresh food such as crispness, color, appearance, taste and sensory attributes [4]. This efficiency was demonstrated through several studies on different fresh products. As an example, Lucera et al., has studied the effect of micro-perforated food packaging on fresh cut cauliflower quality [5]. With regard to ventilated plastic packaging, it is an alternative technique used to improve the packaging permeability. In this technique, it is recommended that the total area of vents should be up to 25% of the packaging area in order to provide sufficient airflow and to increase the permeability of the homogenous air flow inside the packaging [6]. Table 1 summarizes the main characteristics of the MAP and ventilated packaging techniques with advantages, disadvantages, applications and target foods.

In general, porous polymers have recently gained more attention due to the influence of porosity on their properties and functionality. In fact, several methods have been used to create the porosity of the polymeric matrix. These methods include solvent casting/particle leaching, electrospinning and thermally induced phase separation techniques [7]. Indeed, solvent casting is one of the most important techniques that relies on adding a pore forming agent (porogen) into a homogenous polymeric solution and then allowing it to leach out, thus creating pores in its place. Many types of porogen were used in the literature for different applications [8]. Sodium chloride (NaCl) is one the inorganic porogens that could be easily leached out from the polymeric matrix by soaking the membranes in water due to the salt’s high solubility [9,10,11,12]. Combining non-solvent precipitation with sono-crystallization led to control of the nucleation and narrowed the produced size distribution [11,13,14]. To enhance the porous network interconnectivity, water soluble polymeric porogens such as polyethylene oxide (PEO), a hydrophilic thermoplastic polymer, is occasionally combined with inorganic porogens [15,16,17]. Together, they are used as pore forming agent as studied by Reignier et al. [18]. 

Biodegradable polymers have recently gained significant attention in food packaging applications because of their advantages over the petroleum-based polymers due to the adverse effect on the environment by the latter. Polylactic acid (PLA) and polycaprolactone (PCL) are a typical example of these polymers and have found great applications in many food applications. Indeed, barrier properties of PLA are also worth noting. It has a high water vapor transmission rate (WVTR) as compared to other petroleum-based polymers. Also, O_2_TR of PLA is relatively high and its value ranges between the that of terephthalate and polystyrene polymers [19]. Several researchers have investigated the influence of micro-perforated PLA on improving the shelf life of fresh products such as shown by Mistriotis et al. [20]. PCL is also characterized by its flexible chains as well as high elongation and low modulus of elasticity [21]. Nevertheless, low melting point of PCL is considered to be a drawback in many applications and is dealt with by blending PCL with other polymers [22,23]. Rao et al., investigated the mechanical properties of PLA and PCL blends at different compositions [24]. In addition, Scffaro et al., prepared multi-phasic porous laminate from PLA core and PCL shell [25]. Both found great applications of these polymeric blends in food packaging.

From the prospective of food safety, incorporation of an antimicrobial agent to the porous food packaging materials is essential to enhance food protection during storage. In general, antimicrobial food packaging plays a significant role in protecting food from spoilage by inhibiting the microbial growth. Recently, natural food preservatives are sought as alternatives to synthetic preservatives in food preservations [26]. Cinnamaldehyde, a natural antimicrobial active agent, is incorporated in the polymeric membranes as to enhance its antimicrobial properties [27]. 

The aim of this work is to develop environmentally friendly polymeric films based on blends of biodegradable polylactic acid and polycaprolactone polymers for food packaging applications. The microporous films are then coated with cinnamaldehyde to develop antimicrobial breathable films capable of preventing food pathogens from spoiling the packaged food.

## 2. Materials and Methods

### 2.1. Materials 

Poly (lactic acid) (PLA) pellets with a commercial name “Ingeo” grade 4043D were supplied from NatureWorks LLC, MN, USA. The purchased grade has a relative solution viscosity (RV) of 4.0 ± 0.10, and a level of D-isomer of 4.25 ± 0.55%. Polycaprolactone (PCL) pellets were purchased from Sigma-Aldrich, Saint Louis, USA. The purchased grade had an average molecular weight of 80,000 Da. Polyethylene oxide was purchased from Alfa Aesar, Karlusruhe, Germany with molecular weight 100,000 Da. Sodium chloride, absolute ethanol and dichloromethane (DCM) (≤99%) were purchased from Fisher Scientific, Hampton, USA. Trans-cinnamaldehyde was purchased from Scharlau, China.

### 2.2. Preparation Methods

#### 2.2.1. Sodium Chloride Recrystallization

Sodium chloride was recrystallized by using anti-solvent and ultra-sonication techniques. 15 mL of sodium chloride solution (20% weight per water volume) was added to 150 g (192 mL) of absolute ethanol after sonication for 5 min by using ultrasonic probe sonication (Athena Technology, Maharashtra, India) to remove air bubbles. The mixture was exposed to ultra-sonication for 5 min at 132 V under the presence of magnetic stirring to avoid particle agglomeration. The mixture was filtrated under vacuum and the recrystallized particles were obtained. 

#### 2.2.2. Poly (Lactic Acid) (PLA) and Polycaprolactone (PCL)/PLA Films Preparation Method

Samples prepared solely from PLA polymer and from blends of PLA ad PCL of various compositions with different concentrations of the porogenic agent of NaCl and PEO are shown in Table 2 [28]. For all the samples, DCM was used as a solvent and it was added according to the total polymer weight to keep the *w/v* ratio (polymer/solvent) at 10%. PEO and NaCl were also calculated according to the total polymer weight and were added to the 100% polymer base.

The mixture, composed of polyethyleneoxide (PEO), sodium chloride (NaCl), polylacticacid (PLA) and polycaprolactone (PCL), was then sonicated for 1 min at 220 V to ensure complete dispersion of the salt particles. PLA or PCL/PLA blends as well as the PEO were then added to the NaCl/solvent suspended solution and the mixtures were left under magnetic stirring for 2 h at room temperature. The films were cast over glass plates by using automatic film applicator (JR400D, Shanghai, China) and left for 24 h at room temperature to ensure solvent evaporation. The glass plates were then immersed in water to facilitate the removal of the films without cutting or stretching. The films were then immersed in distilled water for 48 days to ensure the removal of all PEO and NaCl crystals. The films then were dried in a vacuum oven at 40° C and 600 mbar for 24 h to ensure the complete evaporation of any solvent and water residuals before characterizations.

### 2.3. Characterization Methods

#### 2.3.1. Scanning Electron Microscopy (SEM)

NaCl crystal size and size distribution as well as the membranes’ morphology were investigated using scanning electron microscope (Supra 5S LEQ, Zeiss). Both the NaCl particles and the membranes were gold spattered for 1min at 15 mA before scanning to avid the effect of electrical charge on the scanned images. 

#### 2.3.2. Air Permeability

Air permeability was measured according to ASTM D 737 standard method by using an air permeability tester, manufactured by TEXTEST Instruments, Switzerland [29]. The measurements were performed at the National Institute of Standards, Egypt. The tested sample was placed on the sample holder and air was drawn under 2125 Pa through an area of 5 cm^2^ of the film. The measurement was replicated three times on different areas of the films and air permeability was taken as an average number in cm^3^/cm^2^/s.

#### 2.3.3. Mechanical Properties Measurement

Maximum elongation, maximum nominal force and energy required to reach maximum elongation were obtained from stress–strain isotherms according to ASTM D822 standard method [30]. Samples with dimensions of 1.5 cm × 9 cm × 0.0007 cm were cut from the films to study the effect of NaCl and blend compositions on its mechanical behavior. Two distinguished lines were drawn on the sample before hanging to determine the elongation versus the stress. The sample was fixed between two clamps and exposed to the tensile force. The upper clamp was fixed while the lower one was connected to a strain gauge. The data were recorded through a digital oscilloscope which was connected to a transducer supplied by a constant voltage DC power supply. The upper clamp was constantly strained and the length between the remarked lines was determined by using a cathetometer to calculate the elongation. Readings from the oscilloscope were recorded for each strain after 15 min in order to ensure the reading stability and the potential was calibrated in Newton (N) from the stress gauge. The elastic force (f) was determined versus elongation and the equilibrium elastic force (f *) was calculated according to the following equation:

f* = f/A*(1)

where f is the elastic force in (N) and A* is the cross-sectional area. The stress–strain graph was then plotted. The maximum energy needed to reach the maximum elongation (E_m_) was determined by calculating the area under the stress–strain curve.

#### 2.3.4. Differential Scanning Calorimetry (DSC)

Differential scanning calorimetry DSC131 evo (SETARAM Inc., Paris, France) was used to perform the differential scanning calorimeter analysis. The samples were weighed in an aluminum crucible of 100 µL and introduced to the DSC. Nitrogen and helium were used as the purging gases. The temperature range was set to 25 °C to 200 °C with a heating rate of 10 °C/min.

#### 2.3.5. Oxygen Transmission Rate (O2TR)

Oxygen transmission rate was measured according to the ASTM D3985 standard method using a GDP-Gas Permeability Tester, Brugger FeinmechaniK Gmbh, Munich, Germany [31]. The sample was fixed between two chambers at ambient atmospheric pressure. The upper chamber was filled with oxygen while the lower chamber was purged with nitrogen, as the carrier gas. Concentration of the oxygen gas permeated through the sample was measured by coulometric detector. The test was run until steady state conditions were obtained. Oxygen transmission rate value was measured in cm^3^/m^2^.d.atm corresponding to the amount of oxygen that permeated through the sample per unit time and area under steady state conditions. 

#### 2.3.6. Water Vapor Transmission Rate (WVTR)

Water vapor transmission rate was measured according to the ASTM E96/E96M-12 standard method [32]. The test was undertaken by using the desiccant method. The desiccant used was calcium chloride CaCl_2_ pre-dried at 200 °C for 24 h. Samples free from pinholes or air bubbles were cut from the cast films in areas of 12.56 cm^2^ and placed inside the autoclave filled with the dried CaCl_2_. The autoclaves were weighed every day (M_t_) for 7 days. WVTR was calculated at the end of each test according to the following equation:

WVTR = (M_t_ − M_0_)/∆t.A (2)

where M_t_ and M_0_ are the sample weights at time t and zero time, respectively, in grams, t is the time in days and A is the tested area of the film in squared meters. 

#### 2.3.7. Water Absorption Test

Water absorption was measured according to the ASTM D570 standard method [33]. Samples were cut in areas of 76 mm × 25 mm. The samples were dried in vacuum oven at 40 °C and 600 mbar for 24 h and then weighed (W_c_). The dried samples were completely immersed in distilled water at 23 °C for 24 h. The samples were then weighed again (W_i_) after removing them from distilled water and wiping them off using tissue papers to remove any excess water from the surface. Percentage of weight gain (W_g_%) was calculated from the difference of W_c_ and W_i_ according to the following equation:

W_g_% = ((W_i_ − W_c_)/W_c_) × 100 (3)

To determine the percentage of weight loss (W_L_%), the samples were re-dried using the same previous conditions and then weighed again (W_f_). Weight loss percentage after re-drying was calculated according to the following equations:

W_L_% = ((W_c_ − W_f_)/W_c_) × 100 (4)

The percentage of water absorption is calculated by simple the addition of the percentage of weight gain and the percentage of weight loss.

#### 2.3.8. Brunauer–Emmett–Teller (BET) Surface Area

Surface area and porosity were determined by nitrogen gas sorption analysis using an ASAP 2020 analyzer (Micromeritics Instrument Corporation, Norcross, GA, USA). The samples were first pretreated at vacuum at 40 °C for 4 h. We used 48-point pressure tables with 20 s equilibration intervals to record the adsorption and desorption isotherms. The surface area was calculated using the method of Brunauer, Emmett, and Teller (BET) by applying a model of adsorption involving multilayer coverage filling. The deBoer t-plot method was carried out to determine the surface area and the micropore volume.

#### 2.3.9. Fourier-Transform Infrared Spectroscopy (FTIR)

FT-IR spectra of the prepared films were investigated to study the interaction between the polymers and the porogen. The spectra were obtained by a Thermo Scientific Nicolet 380 FT-IR, Waltham, MA, USA. The samples were cut into 2 cm × 2 cm and measured under a wavelength range of 500 and 4000 cm^−1^.

#### 2.3.10. Antibacterial Agent Coating

Two samples from each film were cut into 1 cm × 1 cm and immersed in 20 mL ethanol/water mixtures with different concentrations of trans-cinnamaldehyde according to a standard procedures [34]. The molar ratio of ethanol to water in the mixture was 1:4. The samples were kept immersed in the solutions for 24 h and sterilized under an ultraviolet UV lamp to remove any contamination before measuring the antimicrobial activity.

#### 2.3.11. Antibacterial Activity Measurement

An antimicrobial activity test was carried out at the Biochemistry Central Lab, Cairo University, Egypt. The sterilized media were poured onto the sterilized 25 mL Petri dishes and left for solidification at room temperature. Microbial suspension was prepared in sterilized saline equivalent to McFarland 0.5 standard solution (1.5 × 105 CFU mL^−1^). A sterile cotton swab was immersed in the adjusted suspension within 15 min after optimizing the turbidity of the inoculum suspension. The specimens were then flooded on the dried agar surface and left to dry for 15 min. Wells of 6 mm diameter were made in the solidified media using a sterile borer. The films under testing (1 × 1 cm^2^) were put into each well. The plates were incubated at 37 °C for 24 h and zones of inhibition were measured in mm. The experiment was undertaken in triplicate to ensure accuracy.

#### 2.3.12. Statistical Analysis

All experiments were performed three times at the minimum with the obtained data represented as mean standard deviation (SD) or as error bars. Statistical analysis was performed through the application of Dunnett’s test. All tests were calculated using GraphPad Prism Software Version 6.

## 3. Results and Discussion

### 3.1. Membranes Morphologies and Porosity

The non-solvent sono-crystallization technique of (NaCl) showed significant effect on the particles morphology. Indeed, the initial size of (NaCl) before recrystallization ranged from 250 to 500 µm. After recrystallization using ultrasonic and anti-solvent techniques, the crystal size was narrowed to a range of 40 to 120 µm. 

**Figure 1:**
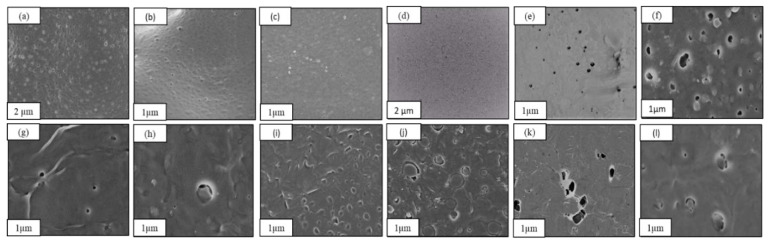
Scanning electron microscopy (SEM) of the macro and micro pores in porous PLA films with different PEO and NaCl percentages. (**a**) PL-N50 (**b**) PL-N70 (**c**) PL-N100 (**d**) PL-PO (**e**) PL-N (**f**) PL-PC2-N50 (**g**) PL-PC3-N50 (**h**) PL-PC4-N50 (**i**) Neat PCL/PLA (4:1) (**j**) PL-PC4-N50 (**k**) PL-PC4-N70 (**l**) PL-PC4-N100.

As shown in Figure 1a–c, all the porous films showed bimodal distribution of macro and micro pores size because of the extraction of NaCl and PEO, respectively [17]. To illustrate further, PEO extraction formed micropores with a size ranging from 200 to 300 nm while NaCl extraction formed macropores with a size of 2 to 8 µm. In fact, small pores act to form interconnected channels between the large pores. This morphology observed for all the porous membranes represents networks composed of large cavities interconnected by small channels. The same result was proved by Reignier et al., and Cui et al., who studied the effect of PEO and NaCl on the porosity of a PCL scaffold [18]. In general, the observed interconnectivity of the porous films is an essential property in enhancing gas transmission rates through packaging materials. Nevertheless, inconsistencies between the crystal size of NaCl alone before mixing with the polymer solution and the size of the macropores formed are worth noting. This unexpected observation indicated that NaCl particles were exposed to cleavage and erosion during the mixing process. Indeed, this phenomenon became more significant at high NaCl percentages as observed in Samples PL-N70 and PL-N100 as the particles started to agglomerate, which affected their size and shape. Furthermore, the porosity should theoretically increase with increasing the NaCl percentage. However, porosity showed an inverse relationship with the NaCl composition. This result was obvious through the high number of interconnected pores observed in the SEM for sample PL-N50 as compared to the small number of pores observed for Samples PL-N70 and PL-N100. This result also emphasizes the agglomeration of NaCl that led to formation of a lower number of pores with relatively larger size as was also concluded from the BET analysis below.

To demonstrate the effect of PEO as a porogenic agent on the porosity, the influence of NaCl and PEO on the porosity was separately investigated. As shown in Figure 1d,e, extraction of 10% PEO in the sample PL-PO led to the formation of a small amount of micropores. However, this amount is much lower than the amount of micropores that were formed with the same percentage of PEO in the presence of NaCl. This observation indicated that the interconnected porous structure resulted from the synergistic effect of both NaCl and PEO. 

Interestingly, the incorporation of PCL into the porous PLA composite has affected the polymeric morphology a great deal as shown in Figure 1j–l in terms of homogeneity, amount of pores and their distribution. Sample PL-PC4-N50 showed the lowest number of small pores compared with samples PL-PC3-N50 and PL-PC2-N50. This observation indicated that the number of small pores decreased with the increase in PCL composition possibly due to the incompatibility between the two polymers resulting in a microphase separation between the two systems. The influence of porogen concentration on the morphology of porous PCL/PLA films was also investigated. As shown in Figure 1j–l, the density of macropores has increased with increasing NaCl percentages signifying a significant extraction of the majority of NaCl particles. Alternatively, the amount of micropores remained low as compared with the PLA films possibly due to the high affinity between PCL and PEO, which led to the incomplete extraction of PEO [29].

### 3.2. Air Permeability

**Figure 2:**
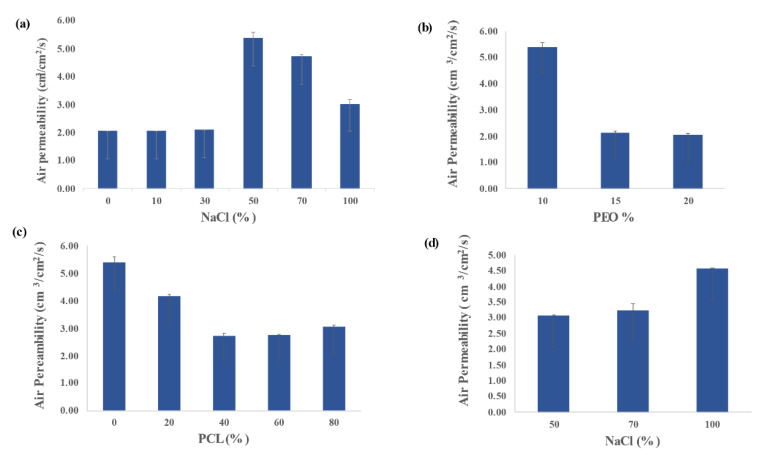
(**a**) The relationship between air permeability and NaCl percentage of porous PLA films. (**b**) The relationship between air permeability and PEO percentage of porous PLA films. (**c**) The relationship between air permeability and PCL percentage of porous PCL/PLA films. (**d**) The relationship between air permeability and NaCl percentage of porous PCL/PLA films.

As shown in Figure 2a below, adding 10% NaCl to Sample PL-N10 did not show any improvement in the air permeability of the film as compared to neat PLA while the sample PL-N30 with 30% NaCl showed slight increase in the air permeability due to the lack of sufficient porosity. On the other hand, the sample PL-N50 showed a significant increase in air permeability when compared to neat PLA. The same observation was noticed for samples PL-N70 and PL-N100 but with lower values than the PL-N50 sample. In fact, the reduction in permeability when NaCl percentage has increased from 50% to 100% could be attributed to the agglomeration of the salt particles, which hinders its leaching and the eventual formation of the macropores as the large salt particles becomes embedded within the polymeric matrices. This result is corroborated further through the BET analysis below as the increase in the pore volume was observed with the increase in the NaCl percentage.

The influence of PEO concentration on the air permeability of porous PLA films was also investigated as shown in Figure 2b. Despite the fact that increasing PEO percentage has improved the NaCl extraction, it has led to a reduction in the air permeability. This result was, in fact, expected due to the influence of the polydispersed pore size and shape distribution on the gas permeability. Consequently, the interconnecting channels that connect the large pores are characterized by high tortuosity, which would naturally lead to an increase in the airflow resistance during gas diffusion as the air diffusion path length increases. Ahmad et al., observed the same inverse proportionality between the tortuosity factor and the air permeability [35]. 

Accordingly, 10% PEO in sample PL-N50 was chosen as the optimal concentration to study the effect of introducing PCL into the polymeric blend on air permeability. The effect of PCL addition is shown in Figure 2c. It is obvious from the figure that samples PL-PC2-N50 and PL-PC3-N50 showed a reduction in air permeability as compared to porous PLA with the same NaCl percentage. This result was expected due to the heterogeneous nature of the blends. In fact, the immiscibility of the two polymers led to the formation of a phase-separated system, which resulted in the decrease of the free volume available for gas diffusion. The influence of NaCl precentage on the air permeability of porous PLA-PCL films was also demonstrated in Figure 2d. The figure shows the increase in the air permeability with the incresae in NaCl concentrations in contrast to that of porous PLA films behavior indicating the complete extraction of well-dispersed NaCl particles as a result of the high affinity of PEO to PCL in good agreement with previous studies [36]. It seems that the phase separation in the PLA/PCL blends has facilitated a high water absorption and consequently enhanced the NaCl solubility and extraction. The small values of the error bars shown in the figures suggest the absence of fluctuation in the air permeability measurements for the different samples indicating high homogeneity of the blends in these samples.

### 3.3. Mechanical Properties

**Figure 3:**
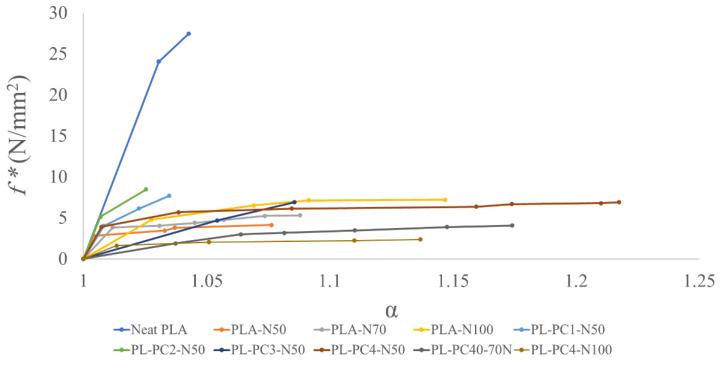
Stress–strain graph of neat PLA and porous PLA and PCL/PLA films.

The effect of NaCl percentage on the mechanical parameters of porous PLA films was investigated. Mechanical properties were discussed based on plotting the stress–strain for each sample. It was found that maximum elongation (α_m_), maximum nominal force (f*_m_) and the maximum energy needed to reach the maximum nominal force and maximum elongation (E_m_) were significantly affected by the developed porosity as well as the remaining NaCl residuals in PLA matrices. As shown in Table 3 and Figure 3, all α_m_, f*_m_ and E_m_ have decreased for all the porous samples when compared to neat PLA. However, the result showed an increase in α_m_, f*_m_ and E_m_ values with the increase in NaCl percentage for Samples PL-N70 and PL-N100 when compared to Sample PL-N50. This result was expected due to the effect of pore volume on resisting deformation.

It was shown previously that porous materials with relative high pore volume can withstand further micro-deformation during stretching [37]. The same result was demonstrated by Buzimov et al., during the study of the effect of zirconia on the mechanical properties of ceramics [38]. Additionally, remaining NaCl particles after leaching acted as a filler and this enhanced the mechanical integrity of the polymeric matrix. This was also demonstrated previously by Roberson et al., during the study of the effect of adding NaCl, as an inorganic filler to a PLA matrix [37].

PCL incorporated into the porous PLA composite has also enhanced its mechanical properties but for samples with at least 80% PCL composition. 

It can be observed from Table 3 and Figure 3 that the samples PL-PC1-N50, PL-PC2-N50 and PL-PC3-N50 did not show improvement in mechanical behavior when compared to the sample PL-N50 while sample PL-PC4-N50 showed a remarked increase in all α_m_, *f*_m_ and E_m_ values. The deteriorative effect of PCL at low concentrations on the mechanical properties is possibly due to the immiscibility and the resultant phase separation of PCL and PLA polymers as was also observed by Takayama et al., while studying a similar impact on polymeric fracture [38]. The influence of NaCl percentage on the mechanical properties of porous PCL/PLA films was different from its effect on porous PLA films. For PCL/PLA blends, α_m_, *f_m_*, and the corresponding E_m_ have decreased with the increase in NaCl percentage as shown in Table 3 and Figure 3. In fact, Gupta et al. demonstrated similar results for porous PCL scaffold [39]. This behavior could be explained on the basis of the formation of brittle structure as the developed pores form weak points within the membrane morphology. As the membrane becomes exposed to tension forces, pores are stretched easily and the tensile strength is reduced. This phenomenon strongly affects the film mechanical properties as the NaCl percentage increases with a parallel increase in the film porosity.

Results of the standard deviation shown in the above table are within acceptable experimental error. Furthermore, slight variation in the measurements may have resulted from the effect of heterogeneous distribution of the pores and the engraved NaCl particles, resulting from the non-complete leaching process.

### 3.4. Differential Scanning Calorimetry Analysis (DSC)

**Figure 4:**
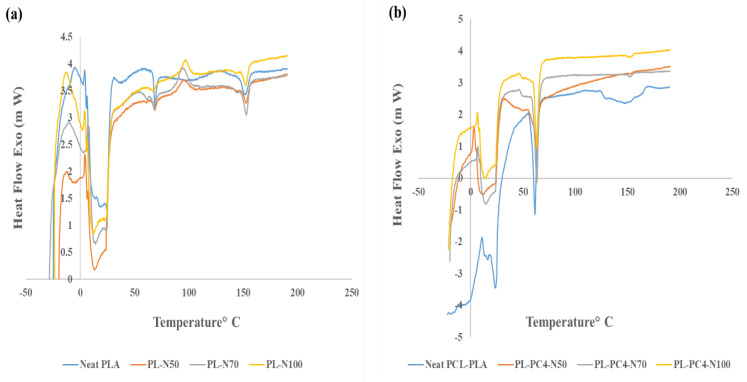
(**a**) Differential scanning calorimetry (DSC) graphs for neat and porous PLA films (**b**) DSC graphs for neat and porous PCL/PLA films.

DSC measurements were taken to investigate the morphology characteristics of the prepared films. As illustrated in Figure 4a and Table 4 below, the results indicate that the porosity did not affect the glass transition behavior (Tg) of the PLA samples. On the other hand, it was observed that the developed porosity had a significant effect on the degree of crystallinity (X_c_%).

Reduction in crystallinity for the sample PL-N50 has resulted from the effect of porosity. To explain, the highly distributed large number of small pores within the confined geometry hindered and interrupted the re-arrangement of the polymeric chains during crystallinity as was also observed by Huang et al., [40]. It is obvious from the results that the direct proportionality of X_c_% and the inverse one of the enthalpy of melting (∆H_m_) with NaCl percentage has resulted from the effect of the agglomerated particles on the pore size in good agreement with the literature with regards to investigating the influence of inorganic fillers on the crystallinity degree [41,42]. For PCL/PLA membranes, high crystallinity was observed as expected since PCL, the major component in the blend, is a semi-crystalline polymer [43]. Interestingly, two crystallization peaks were observed in the DSC thermograms of porous PCL/PLA samples. The first peak appeared with low intensity at 50 °C corresponding to PEO crystallization while the second broad peak appearing at 60 °C corresponded to PCL crystallization as was observed previously by Huang et al., while studying the effect of using PEO as a porogenic agent on PCL crystallinity [44].

Porosity of the blends also had a notable effect on its crystallinity. Sample PL-PC4-N50 showed a reduction in crystallinity as compared to neat PCL-PAL, which is the result of porosity in restricting chain movement [45,46].

### 3.5. Oxygen Transmission Rates (O_2_TR)

Diffusion of gases through the pores depends on the distribution, shape, number and interconnectivity of pores. As illustrated in Table 5, the sample PL-N50 showed the highest O_2_TR due to the high number of interconnected pores followed by the samples PL-N70 and PL-N100. As expected, reduction in convective gas flow in the samples PL-N70 and PL-N100 as compared to sample PL-N50 resulted from the decrease in the number of pores but with an increase in their volume. Increasing PLA crystallinity was also accompanied with a reduction in oxygen permeability. Cougneau et al., demonstrated that reduction in diffusivity and solubility coefficients of oxygen was associated with the increase in PLA crystallinity [47]. Interestingly, O_2_TR of porous PCL/PLA membranes increased with the increase in NaCl percentage with the sample PL-PC4 N100 showing the highest O_2_TR value followed by samples PL-PC4 N70 and PL-PC4 N50 as a result of the formation of a large number of pores.

Importantly, all the samples showed O_2_TR values that are within the acceptable range of O_2_TR for breathable packaging material for several fruits and vegetables as a result of an improvement in the weight loss, chlorophyll content and color of the food products, which was observed in response to the film’s ability to modify the atmospheric composition within the package [48,49].

### 3.6. Water Vapor Transmission Rate (WVTR)

Water vapor transmission rate is another important parameter in food packaging development since water vapor is a major product of the fresh food respiration reaction. 

Packaging film must be permeable for water vapor as, otherwise, food deterioration and microbial growth would occur. Table 6 lists the water vapor transmission rate (WVTR) for the developed films. As is obvious in the table, Sample PL-N50 showed the highest value when compared to samples PL-N70 and PL-N100 as a result of the high distribution of the small pores within the polymeric matrix. Additionally, water solubility within the polymer matrix was also reduced with the increase in the degree of crystallinity since the water solubility in the crystal region is naturally lower than that in the amorphous region [50]. 

The effect of blending PLC with PLA on WVTR was also demonstrated. Slight reduction in WVTR was observed for neat PCL/PLA as compared with neat PLA. Although the diffusion coefficient of water vapor in PCL is higher than that of PLA, PCL is slightly more hydrophobic than PLA, which will affect the water solubility [51]. The immiscibility of PCL and PLA polymers also resulted in the reduction of the free volume and diffusion path available for water diffusion, which consequently reduced WVTR [52]. WVTR values increased for porous PCL/PLA membranes with the increase in NaCl percentage as water vapor transferring through the pores was more predominant than that through the polymer matrix.

### 3.7. Water Absorption Test

As shown in Table 7, a significant increase in water absorption was observed in porous PLA membranes as compared with the neat PLA. These results indicate high capability of the porous film to uptake water and to hold it within its pore cavities due to the availability of high surface area and free volume.

Although there was a reduction in permeability of samples PL-N70 and PL-N100, higher water absorption percentages were observed compared to sample PLA-N50 due to the increase in the films’ surface area and pore volume associated with the increase in NaCl percentage [53]. In fact, this phenomenon is enhanced with high NaCl percentage as a result of the interaction between the solvated cations and the adjacent ions of NaCl residuals within the polymeric matrix [54,55,56]. No significant difference was observed in the water uptake for porous PCL/PLA films.

Values of the standard deviation in the above table are within the acceptable experimental error and the observed slight variation resulted from the heterogeneous nature of the polymeric matrices. Indeed, as mentioned above, the heterogeneity appeared to be due to the non-complete leaching of NaCl particles and the pore distribution in the porous PLA films. In addition, the incompatibility of PLA and PCL polymers contribute largely to the heterogeneity of the porous PCL-PLA films.

### 3.8. Fourier-Transform Infrared Spectroscopy (FTIR) Analysis

FTIR spectra for neat PLA showed sharp peaks at 3685 and 3504.6 cm^−1^, which indicate O–H vibrational stretching mode. The symmetric and asymmetric stretching vibrational modes shown at 2945.3 and 2995.9 cm^−1^ were assigned to the C–H bond in CH_3_ group respectively. The symmetric and asymmetric bending modes of CH_3_ were shown at 1456.4 and 1366.3 cm^−1^, respectively. Stretching vibrational mode of C–O–C for the ester bond was shown at 1132.5, cm^−1^ while stretching vibrational mode of C=O in the ester group was shown at 1781.9 cm^−1^. Furthermore, peaks that appeared at 956 and 894.7 cm^−1^ may represent the stretching vibration of the ester bond (O–C=O) [57,58,59,60].

Moreover, PEO did not form chemical bonds with the functional groups of PLA. Nevertheless, reduction in the intensities of some peaks was observed in the FTIR spectra of the porous samples. This reduction became more significant when NaCl percentage increased in the samples PLA-N70 and PL-N100. In studying the effect of NaCl as a porogenic agent, on FTIR spectra of porous PLA, Sibambo et al. [54], indicated that this interaction resulted from the crosslinking of lactic units within the PLA matrix, which led to reduction in the intensities and broadness of some peaks in its FTIR spectra. In general, FTIR spectra of neat PCL/PLA exhibited the same structure of neat PLA since both polymers are aliphatic polyesters with similar functional groups. FTIR spectra of the blend confirmed the immiscibility of PCL and PLA due to the lack of interaction between the functional groups of the two polymers [61].

### 3.9. Brunauer–Emmett–Teller (BET) Analysis

The impact of different PEO percentages on the surface area and pore volume of porous PLA films was demonstrated through BET analysis.

**Figure 5:**
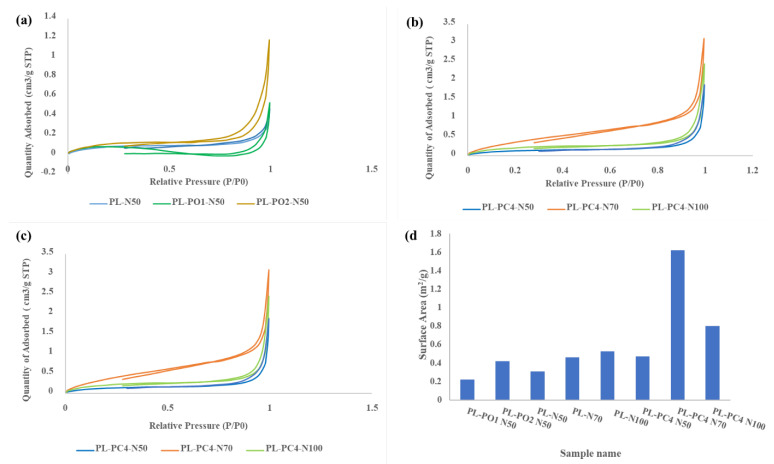
The hysteresis loops for porous PLA and porous PCL/PLA films (**a**) porous PLA with different percentages of PEO (**b**) porous PLA films with different percentages of NaCl (**c**) porous PCL/PLA films with different percentages of NaCl (**d**) the Brunauer–Emmett–Teller (BET) surface area of porous PLA and porous PCL/PLA films.

As shown in Figure 5a, the isotherm for all the samples showed type IV hysteresis. Appearance of this type of hysteresis indicated the existence of pores in the nanoscale and microscale. Moreover, the hysteresis was classified as H4, which indicates the existence of slit pores, internal voids, broad size distribution and heterogeneous porosity. In fact, BET surface area did not show an obvious proportional relationship with PEO percentage. However, the sample PL-PO2-N50 with 20% PEO showed relatively high BET surface area than other samples with lower PEO concentrations. To clarify, BET surface area increased with the increase in the total pore volume. Increasing PEO concentration enhanced NaCl leaching and consequently led to the formation of high numbers of macropores [62]. Figure 5b isotherms also showed type VI hysteresis but classified as H3, which indicated the existence of interconnected pores with uniform channels [63]. The BET surface area and pore volume increased in samples PL-N70 and PL-N100 over that of sample PL-N50. This increase was due to the increasing in crystallinity since crystalline structures enhance the stability of the pores and void its collapse [64,65]. The effect of NaCl percentage on the porosity of porous PCL/PLA films was also investigated through the adsorption isotherms, which showed Type IV as illustrated in Figure 5c. This result indicated that gas adsorption followed a similar pattern for porous PLA. The BET surface areas of porous PCL/PLA films increased with increasing NaCl percentage. This result was expected due to complete leaching of NaCl and the associated developed porosity, which provided high free volume. As shown in Figure 5d, BET analysis showed that the surface areas of porous PCL/PLA films were higher than the surface area of porous PLA films. Accordingly, this contradiction emphasized that the affinity of PEO to PCL hindered NaCl agglomeration and enhanced its full leaching.

### 3.10. Antimicrobial Activity

As shown in Table 8, unlike sample PL-N50, sample PL-PC4-N50 showed antibacterial activity at lower cinnamaldehyde concentration, which indicated that the interaction between cinnamaldehyde and PL-PC4-N50 films was stronger than its interaction with PLA ones possibly due to the hydrophobicity of PCL over PLA, as was also reported by Patel et al., for the hydrophobic cucurbitacin drug loading into a PCL-PEO diblock copolymer [66]. Several studies targeting the design of new drug-delivery systems showed that incorporating PEO into PCL matrix facilitated the penetration of the aqueous solution into the hydrophobic matrix [67]. In general, for the two samples, the inhibition zones against *Staphylococcus aureus* (*S. aureus*) were higher than the inhibition zones against *Escherichia coli* (*E. coli*). 

**Figure 6:**
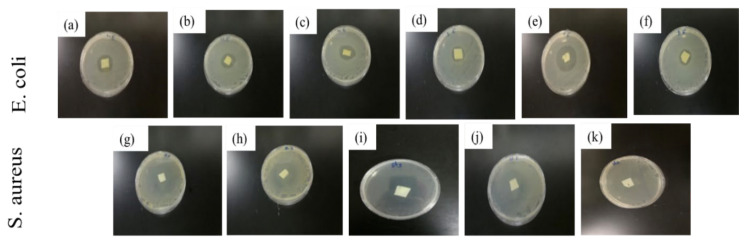
Inhabitation zones of porous PLA and porous PCL/PLA films soaked in cinnamaldehyde. (**a**) PL-N50 (**b**)PL-N70 (**c**) PL-N100 (**d**) PL-PC4-N50 (**e**) PL-PC4-N70 (**f**) PL-PC4 N100 (**g**) PL-N50 (**h**) PL-N70 (**i**) PL-PC4-N50 (**j**) PL-PC4-N70 (**k**) PL-PC4 N100.

This result, Figure 6, was expected since the structure of the outer membrane for the two bacteria is different [68]. Cinnamaldehyde concentration of 6% did not show antimicrobial effect against either *E. coli* or *S. aureus* for all the porous PLA samples as is obvious from Table 8. However, at the same concentration, antimicrobial activity against *S. aureus* was shown with all porous PCL/PLA films. Indeed, the inhibition zones of the PL-PC4-N70 and PL-PC-N100 samples were higher that the inhibition zone shown for the sample PL-PC4-N50 due to the effect the porosity on enhancing the antimicrobial loading capacity and the antimicrobial activity. Nevertheless, at the same concentration, only the PL-PC4-N70 sample showed an inhibition zone of 22 mm against *E. coli* due to its higher surface area. Moreover, soaking the solution with cinnamaldehyde concentration of 7% showed a significant increase in the inhibition zones of samples PL-N70 and PL-N100 against *S. aureus* as compared to sample PL-N50. This result indicated that the loading capacity for cinnamaldehyde within Samples PL-N70 and PL-N100 was higher than for the sample PL-N50 as a result of the high surface area. For the same reason, these samples started to show antimicrobial activity against *E. coli* at this concentration. In addition, the antimicrobial activity of all the porous PCL/PLA films was enhanced at this concentration against both *S. aureus* and *E. coli*.

Increasing the cinnamaldehyde concentration from 7% to 8% led to the enhancement of the antimicrobial properties of the sample PL-N50 against *E. coli*. Interestingly, the other porous PLA and PCL/PLA samples showed a reduction or no significant change in the inhibition zones at this concentration. As the cinnamaldehyde concentration increased to 8%, no obvious proportional relationship was observed between the size of the inhibition zones and NaCl percentages. It is obvious that the distribution of cinnamaldehyde has become uneven at this concentration due to its hydrophobic nature leading to an inhomogeneous coating of the films.

## 4. Conclusions and Future Work

The purpose of this work was to develop antimicrobial breathable films from biodegradable polymers for food packaging applications. According to this study, all developed samples achieved O_2_TR values within the acceptable range for breathable food packaging with different mechanical properties. Indeed, sample PL-N50 and sample PL-PC4-N50 showed the highest air permeability and O_2_TR values with acceptable mechanical properties. However, sample PL-N50 was characterized as having low elongation values. However, sample PL-PC4-N50 showed acceptable mechanical properties and relatively high barrier properties.

**Figure 7:**
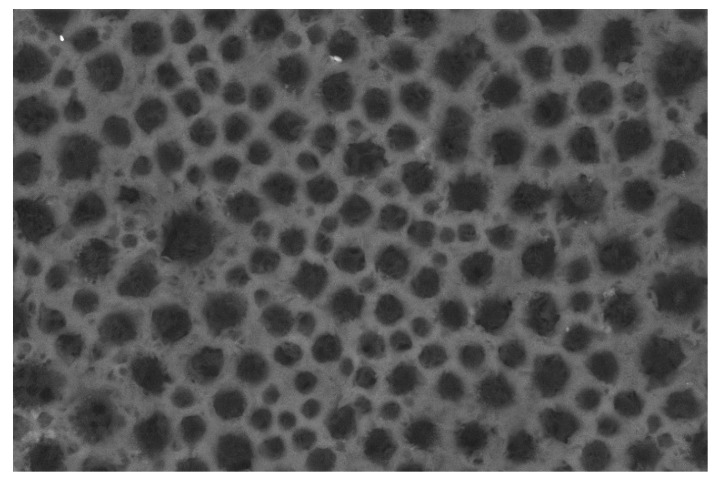
Synthesis of polymeric membranes with intrinsic microporosity.

While this work focused only on the development of porous biodegradable polymeric membrane for food packaging applications through extrinsic micro-porosity approach, using in situ intrinsic micro/nano-porosity through chemical synthesis methods was also considered [4]. Accordingly, contorted 5,5’,6,6’-tetrahydroxy-3,3,3’,3’ tetramethyl-1,1’-spirobisindane monomer is currently being examined as a possible starting material in this approach to create polymeric membranes of intrinsic microporosity, as shown in Figure 7 [48]. 

## Figures and Tables

**Table 1:** Summary of the main characteristics of perforated modified atmospheric packaging (MAP) and ventilated food packaging.

	Perforated Modified Atmospheric Packaging (MAP)	Ventilated Food Packaging
**Main principle**	Modifying the compositions of air gases within the packaging headspace to an optimum ratio as to increase the shelf life of the packaged food [1,2].	Providing channels for air flow within the packaging material to facilitate air exchange between the cooling medium and the product inside the packaging [6,7].
**Advantages**	MAP is considered an important technique for increasing the shelf life of perishable products without using high amount of chemical preservatives [1].	Orovides for an appropriate flow rate that reduces water vapor deficit to prevent fruits decay and consumed cooling energy [8].
**Disadvantages**	Macro-perforated food packages contain large holes unable to modify the atmospheric gases. Microporous films can regulate the gas exchange and reduce moisture loss, which may lead to an increase in water vapor concentration that encourages microbial growth [3].	Inefficient ventilated packaging leads to heterogeneous airflow during cooling and creating cooling gradients, which leads to fast cooling of perimeter foods followed by slower cooling of foods placed in the middle and to the back of the cooling source, respectively [8].
**Applications**	MAP is used to preserve crops characterized with high respiration rates to increase fresh food preservation [4,5].	Ventilated packaging is used to improve the cooling process efficiency during storage of food packaging applications [7,9].

**Table 2:** Porous Poly (lactic acid) (PLA) and PLA/polycaprolactone (PCL) films with different porogenic concentrations.

PLA Sample	Porogenic Agents Compositions		PLA/PCL Sample	Polymer Base Compositions		Porogenic Agents Compositions	
	PEO %	NaCl %		PLA %	PCL %	PEO %	NaCl %
Neat PLA	0	0	Neat PL-PLA	80	20	0	0
PL-PO	10	0	PL-PO2-N50	100	0	20	50
PL-N	0	50	PL-PC1-N50	80	20	10	50
PL-N10	10	10	PL-PC2-N50	60	40	10	50
PL-N30	10	10	PL-PC3-N50	40	60	10	50
PL-N50	10	50	PL-PC4-N50	20	80	10	50
PL-N70	10	70	PL-PC4-N70	20	80	10	70
PL-100	10	100	PL-PC4-N100	20	80	10	100
PL-PO1-N50	15	50					

**Table 3:** Maximum elongation, nominal force and maximum energy of the various films.

Sample Name	α_m_	f_m_* (*N*mm^−2^)	E_m_ (J mm^−1^)	Sample Name	α_m_	f_m_* (*N*mm^−2^)	E_m_ (J mm^−1^)
Neat PLA	1.13 ± 0.08	27.54 ± 1.35	3.1 ± 0.19	PL-PC2-N50	1.03 ± 0.05	8.45 ± 0.37	0.24 ± 0.02
PL-N50	1.08 ± 0.15	4.1 ± 0.28	1.08 ± 0.07	PL-PC3-N50	1.09 ± 0.07	6.89 ± 0.42	0.33 ± 0.02
PL-N70	1.09 ± 0.07	5.35 ± 0.39	1.1 ± 0.06	PL-PC4-N50	1.22 ± 0.06	6.78 ± 042	1.31 ± 0.08
PL-N100	1.15 ± 0.06	7.18 ± 0.45	1.15 ± 0.08	PL-PC4-N70	1.17 ± 0.07	3.36 ± 0.22	0.61 ± 0.04
PL-PC1-N50	1.03 ± 0.05	7.67 ± 0.42	0.13 ± 0.01	PL-PC4-N100	1.14 ± 0.05	2.35 ± 0.10	0.33 ± 0.02

**Table 4:** Thermal properties of neat and porous PLA and PCL/PLA films.

Sample Name	Tg °C	Tcc °C	Tm °C	∆Hm PLA (J/g)	Xc (%) PLA	Xc (%) PCL
Neat PLA	67.45	NA	151.55	17.73	19.06	NA
PL-N50	68.83	96.55	152.91	9.83	10.57	NA
PL-N70	68	94.48	152.41	14.44	15.53	NA
PL-N100	67.55	96.43	152.7	18.21	19.58	NA
Neat PCL/PLA	NA	NA	61.78	68.85	NA	61.69
PL-PC4-N50	NA	NA	61.67	41.36	NA	37.06
PL-PC4-N70	NA	NA	64.02	73.68	NA	66.02
PL-PC4-N100	NA	NA	65.1	52.49	NA	47.03

**Table 5:** Oxygen transmission rates (O_2_TR) of neat PLA and porous PLA and PCL/PLA films.

Sample Name	O_2_TR (c.c/m2.d.atm)	Increasing in O_2_TR (%)	Sample Name	O_2_TR (c.c/m2.d.atm)	Increasing in O_2_TR (%)
Neat PLA	950	_	PL-PC4-N50	3210	237.89
PL-N50	4540	377.89	PL-PC4-N70	4040	325.26
PL-N70	3920	312.63	PL-PC4-N100	4540	377.89
PL-N100	3040	220.00			

**Table 6:** Water vapor transmission rate (WVTR) of neat PLA and porous PLA and PCL/PLA films.

Sample Name	WVTR (g/d.m^2^)	Sample Name	WVTR (g/d.m^2^)
Neat PLA	78.93 ± 1.63	Neat PCL-PAL	73.73 ± 0.95
PL-N50	327.73 ± 0.53	PL-PC4 N50	304.53 ± 7.31
PL-N70	324.13 ± 7.6	PL-PC4 N70	318.52 ± 5.29
PL-N100	315.73 ± 4.08	PL-PC4 N100	334.93 ± 7.35

**Table 7:** Water absorption and weight loss of neat and porous PLA and PCL/PLA films.

Sample Name	Weight Loss (%)	Water Absorption (%)	Sample Name	Weight Loss (%)	Water Absorption (%)
Neat PLA	0.5 ± 0.01	0.6 ± 0.02	Neat PCL/PLA	0.8 ± 0.02	0.9 ± 0.04
PL-N50	14.8 ± 0.57	31.1 ± 0.37	PL-PC4-N50	9.0 ± 0.25	17.8 ± 0.33
PL-N70	16.2 ± 0.32	35.3 ± 0.33	PL-PC4-N70	12.8 ± 0.41	26.2 ± 0.29
PL-N100	19.2 ± 0.49	42.5 ± 0.45	PL-PC4-N100	22.1 ± 0.49	50.7 ± 0.53

**Table 8:** Inhibition zones of porous PLA and PCL/PLA films for *Staphylococcus aureus* and *Esherichia coli* with different cinnamaldehyde concentrations.

Cinnamaldahyde (%)	Inhibition Zone with *S. aureus* (mm)						Inhibition Zone with *E. coli* (mm)					
	PL-N50	PL-N70	PL-N100	PL-PC4-N50	PL-N70	PL-PC4-N100	PL-N50	PL-N70	PL-N100	PL-PC4-N50	PL-PC4-N70	PL-PC4-N100
6	NA	NA	NA	15 ± 0.2	40 ± 0.3	34 ± 0.2	NA	NA	NA	NA	22 ± 0.1	NA
7	14.3 ± 1	45 ± 0.5	47 ± 0.5	38 ± 0.5	40 ± 0.4	27 ± 0.2	NA	24 ± 0.1	23 ± 0.2	22 ± 0.1	37 ± 0.4	19 ± 0.3
8	35 ± 0.3	29 ± 0.2	35 ± 0.4	33 ± 0.4	40 ± 0.3	NA	25 ± 0.5	28 ± 0.5	24 ± 0.5	22 ± 0.4	22 ± 0.5	NA

## Data Availability

Data and experimental information used to produce this manuscript or needed to reproduce the work reported herein are all available in the various sections of this manuscript.

## References

[1] Becker B.R., Fricke B.A. (1996). Transpiration and respiration of fruits and vegetables. Sci. Tech. Froid.

[2] Alfei S., Barbara M., Guendalina Z. (2020). Nanotechnology application in food packaging: A plethora of opportunities versus pending risks assessment and public concerns. Food Res. Int..

[3] Sandhya (2010). Modified atmosphere packaging of fresh produce: Current status and future needs. LWT-Food Sci. Technol..

[4] Elmehalmey W.A., Azzam R.A., Hassan Y.S., Alkordi M.H., Madkour T.M. (2018). Imide-based polymers of intrinsic microporosity: Probing the microstructure in relation to CO_2_ sorption characteristics. ACS Omega.

[5] Lucera A., Conte A., Del Nobile M.A. (2012). Shelf Life of Ready-to-Cook Cauliflower Mixtures as Affected by Packaging Film Mass Transport Properties: Ready-to-Cook Cauliflower Mixture. Int. J. Food Sci. Technol..

[6] Berry T.M., Fadiji T.S., Defraeye T., Opara U.L. (2017). The role of horticultural carton vent hole design on cooling efficiency and compression strength: A
multi-parameter approach. Postharvest Biol. Technol..

[7] El-Sayed M.M., Elsayed R.E., Attia A., Farghal H.H., Azzam R.A., Madkour T.M. (2021). Novel nanoporous membranes of bio-based cellulose acetate, poly (lactic acid) and biodegradable polyurethane in-situ impregnated with catalytic cobalt nanoparticles for the removal of Methylene Blue and Congo Red dyes from wastewater. Carbohydr. Polym. Technol. Appl..

[8] Mane S. (2016). Effect of Porogens (Type and Amount) on Polymer Porosity: A Review. Can. Chem. Trans..

[9] Dorati R., Colonna C., Genta I., Modena T., Conti B. (2010). Effect of porogen on the physico-chemical properties and degradation performance of PLGA scaffolds. Polym. Degrad. Stab..

[10] Tran R.T., Naseri E., Kolasnikov A., Bai X., Yang J. (2011). A New Generation of Sodium Chloride Porogen for Tissue Engineering. Biotechnol. Appl. Biochem..

[11] Lee J., Ashokkumar M., Kentish S.E. (2014). Influence of mixing and ultrasound frequency on antisolvent crystallisation of sodium chloride. Ultrason. Sonochem..

[12] Gielen B., Jordens J., Thomassen L., Braeken L., Van Gerven T. (2017). Agglomeration control during ultrasonic crystallization of an active pharmaceutical ingredient. Crystals.

[13] Takiyama H., Otsuhata T., Matsuoka M. (1998). Morphology of NaCl crystals in drowning-Out precipitation operation. Chem. Eng. Res. Des..

[14] Abbas A., Srour M., Tang P., Chiou H., Chan H.-K., Romagnoli J.A. (2007). Sonocrystallisation of sodium chloride particles for inhalation. Chem. Eng. Sci..

[15] Mi H.-Y., Jing X., Turng L.-S. (2015). Fabrication of porous synthetic polymer scaffolds for tissue engineering. J. Cell. Plast..

[16] Bailey F.E. (2012). Poly (Ethylene Oxide).

[17] Madkour T.M., Hamdi M.S. (1996). Elastomers with two crosslinking systems of different lengths viewed as bimodal networks. J. Appl. Polym. Sci..

[18] Reignier J., Huneault M.A. (2006). Preparation of interconnected Poly(ε-Caprolactone) porous scaffolds by a combination of polymer and salt particulate leaching. Polymer.

[19] Yuniarto K., Welt B.A., Purwanto A., Purwadaria H.K., Abdellatief A., Sunarti T.C., Purwanto S. (2014). Effect of plasticizer on oxygen permeability of cast polylactic acid (PLA) films determined using dynamic accumulation method. J. Appl. Packag. Res..

[20] Mistriotis A., Briassoulis D., Giannoulis A., D’Aquino S. (2016). Design of Biodegradable Bio-Based Equilibrium Modified Atmosphere Packaging (EMAP) for Fresh Fruits and Vegetables by Using Micro-Perforated Poly-Lactic Acid (PLA) Films. Postharvest Biol. Technol..

[21] Dasari A., Njuguna J. (2016). Functional and Physical Properties of Polymer Nanocomposites.

[22] Labet M., Thielemans W. (2009). Synthesis of Polycaprolactone: A Review. Chem. Soc. Rev..

[23] Guarino V., Gentile G., Sorrentino L., Ambrosio L. (2017). Polycaprolactone: Synthesis, Properties, and Applications.

[24] Rao R.U., Suman K., Rao V.K., Bhanukiran K. (2011). Study of Rheological and Mechanical Properties of Biodegradable Polylactide and Polycaprolactone Blend. Int. J. Eng. Sci. Technol..

[25] Scaffaro R., Lopresti F., Botta L., Maio A. (2016). Mechanical Behavior of Polylactic Acid/Polycaprolactone Porous Layered Functional Composites. Compos. Part B Eng..

[26] Atarés L., Chiralt A. (2016). Essential Oils as Additives in Biodegradable Films and Coatings for Active Food Packaging. Trends Food Sci. Technol..

[27] Simona J., Dani D., Petr S., Marcela N., Jakub T., Bohuslava T. (2021). Edible films from carrageenan/orange essential oil/trehalose—structure, optical properties, and antimicrobial activity. Polymers.

[28] Mohamed M. (2019). Design and Development of Biodegradable Microporous Polymeric Systems with Enhanced Characteristics for Food Packaging Applications. Master’s Thesis.

[29] ASTM International ASTM D737-18 Standard Test Method for Air Permeability of Textile Fabrics. https://www.astm.org/Standards/D737.htm.

[30] ASTM International ASTM D882-18 Standard Test Method for Tensile Properties of Thin Plastic Sheeting. https://www.astm.org/Standards/D882.

[31] ASTM International ASTM D3985-17 Standard Test Method for Oxygen Gas Transmission Rate Through Plastic Film and Sheeting Using a Coulometric Sensor. https://www.astm.org/Standards/D3985.htm.

[32] ASTM International ASTM E96 / E96M-12 Standard Test Methods for Water Vapor Transmission of Materials. https://www.astm.org/DATABASE.CART/HISTORICAL/E96E96M-12.htm.

[33] ASTM International ASTM D570–98 (2018) Standard Test Method for Water Absorption of Plastics. https://www.astm.org/Standards/D570.

[34] Bierhalz A.C.K., da Silva M.A., de Sousa H.C., Braga M.E.M., Kieckbusch T.G. (2013). Influence of Natamycin Loading Methods on the Physical Characteristics of Alginate Active Films. J. Supercrit. Fluids.

[35] Ahmad S., Azad A.K., Loughlin K.F. (2005). A study of permeability and tortuosity of concrete. Proceedings of the 30th Conference on Our World in Concrete and Structures.

[36] Madkour T.M., Azzam R.A., Mark J.E. (2006). Recent advances in the modeling and simulation of metallocene catalysis, sequence distribution, chain conformations, and crystallization of polymers. J. Polym. Sci. B Polym. Phys..

[37] Ibrahim A.H., Zikry A.A., Madkour T.M. (2017). Enhanced thermal stability of “environmentally friendly” biodegradable poly (Lactic acid) blends with cellulose acetate. an experimental and molecular modeling study. Biointerface Res. Appl. Chem..

[38] Buzimov A.Y., Kulkov S.N., Eckl W., Pappert S., Gömze L.A., Kurovics E., Kocserha I., Géber R. (2017). Effect of Mechanical Treatment on Properties of Zeolites with Chabazite Structure. J. Phys. Conf. Ser..

[39] Gupta B., Patra S., Ray A.R. (2012). Preparation of Porous Polycaprolactone Tubular Matrix by Salt Leaching Process. J. Appl. Polym. Sci..

[40] Huang R., Zhu X., Tu H., Wan A. (2014). The Crystallization Behavior of Porous Poly(Lactic Acid) Prepared by Modified Solvent Casting/Particulate Leaching Technique for Potential Use of Tissue Engineering Scaffold. Mater. Lett..

[41] Madkour T.M., Mark J.E. (2002). Mesoscopic modeling of the polymerization, morphology, and crystallization of stereoblock and stereoregular polypropylenes. J. Polym. Sci. B Polym. Phys..

[42] Madkour T.M., Abdelazeem E.A., Tayel A., Mustafa G., Siam R. (2016). In situ polymerization of polyurethane-silver nanocomposite foams with intact thermal stability, improved mechanical performance, and induced antimicrobial properties. J. Appl. Polym. Sci..

[43] Madkour T.M., Azzam R.A. (2002). Use of blowing catalysts for integral skin polyurethane applications in a controlled molecular architectural environment: Synthesis and impact on ultimate physical properties. J. Polym. Sci. A Polym. Chem..

[44] Huang A., Jiang Y., Napiwocki B., Mi H., Peng X., Turng L.S. (2017). Fabrication of poly (ε-caprolactone) tissue engineering scaffolds with fibrillated and interconnected pores utilizing microcellular injection molding and polymer leaching. RSC Adv..

[45] Yin G., Zhang L., Zhou Z., Li Q. (2016). Preparation and Characterization of Cross-Linked PCL Porous Membranes. J. Polym. Res..

[46] Qiu Z., Ikehara T., Nishi T. (2003). Miscibility and Crystallization of Poly (Ethylene Oxide) and Poly(ε-Caprolactone) Blends. Polymer.

[47] Courgneau C., Domenek S., Lebossé R., Guinault A., Avérous L., Ducruet V. (2012). Effect of crystallization on barrier properties of formulated polylactide. Polym. Int..

[48] Sabaa M.W., Madkour T.M., Yassin A.A. (1988). Polymerization products of p-benzoquinone as bound antioxidants for SBR. Part II—The antioxidizing efficiency. Polym. Degrad. Stab..

[49] Pan X.C., Sasanatayart R. (2016). Effect of plastic films with different oxygen transmission rate on shelf-life of fresh-cut bok choy (Brassica rapa var. chinensis). Int. Food Res. J..

[50] Duan Z., Thomas N.L. (2014). Water Vapour Permeability of Poly(Lactic Acid): Crystallinity and the Tortuous Path Model. J. Appl. Phys..

[51] Ikada Y., Tsuji H. (2000). Biodegradable polyesters for medical and ecological applications. Macromol. Rapid Commun..

[52] Bouakaz B.S., Habi A., Grohens Y., Pillin I. (2017). Organomontmorillonite/Graphene-PLA/PCL Nanofilled Blends: New Strategy to Enhance the Functional Properties of PLA/PCL Blend. Appl. Clay Sci..

[53] Nasri-Nasrabadi B., Mehrasa M., Rafienia M., Bonakdar S., Behzad T., Gavanji S. (2014). Porous starch/cellulose nanofibers composite prepared by salt leaching technique for tissue engineering. Carbohydr. Polym..

[54] Sibambo S.R., Pillay V., Choonara Y.E., Penny C. (2008). A Novel Salted-out and Subsequently Crosslinked Poly(Lactic-Co-Glycolic Acid) Polymeric Scaffold Applied to Monolithic Drug Delivery. J. Bioact. Compat. Polym..

[55] Elsayed R.E., Madkour T.M., Azzam R.A. (2020). Tailored-design of electrospun nanofiber cellulose acetate/poly (lactic acid) dressing mats loaded with a newly synthesized sulfonamide analog exhibiting superior wound healing. Int. J. Biol. Macromol..

[56] Sánchez-González S., Diban N., Urtiaga A. (2018). Hydrolytic degradation and mechanical stability of poly (ε-Caprolactone)/reduced graphene oxide membranes as scaffolds for in vitro neural tissue regeneration. Membranes.

[57] Mkhabela V.J., Ray S.S. (2015). Fabrication of polylactide nanocomposite scaffolds for bone tissue engineering applications. AIP Conference Proceedings.

[58] Lasprilla A.R., Martinez G.R., Hoss B. (2011). Synthesis and characterization of poly (lactic acid) for use in biomedical field. Chem. Eng..

[59] Madkour T.M., Azzam R.A. (2013). Non-Gaussian behavior of self-assembled thermoplastic polyurethane elastomers synthesized using two-step polymerization and investigated using constant-strain stress relaxation and molecular modeling techniques. Eur. Polym. J..

[60] Fadl S.M. (2015). Development and Characterization of Biodegradable Biorenewable Polymeric Nanocomposites for Food Packaging Applications. Master’s Thesis.

[61] Mekewi M.A., Madkour T.M., Darwish A.S., Hashish Y.M. (2015). Does poly (acrylic acid-co-acrylamide) hydrogel be the pluperfect choiceness in treatment of dyeing wastewater? “From simple copolymer to gigantic aqua-waste remover”. J. Ind. Eng. Chem..

[62] Wang H., Min S., Ma C., Liu Z., Zhang W., Wang Q., Li D., Li Y., Turner S., Han Y. (2017). Synthesis of single-crystal-like nanoporous carbon membranes and their application in overall water splitting. Nat. Commun..

[63] Azzam R.A., Madkour T.M. (2021). Synthesis of Novel Bio-based Urea-Urethane Aerogels In-Situ Impregnated with Catalytic Metallic Nanoparticles for the Removal of Methylene Blue and Congo Red from Wastewater. J. Polym. Environ..

[64] Livshin S., Silverstein M.S. (2008). Crystallinity and cross-linking in porous polymers synthesized from long side chain monomers through emulsion templating. Macromolecules.

[65] Nuhnen A., Dietrich D., Millan S., Janiak C. (2018). Role of filler porosity and filler/polymer interface volume in metal–organic framework/polymer mixed-matrix membranes for gas separation. ACS Appl. Mater. Interfaces.

[66] Patel S.K., Lavasanifar A., Choi P. (2009). Roles of nonpolar and polar intermolecular interactions in the improvement of the drug loading capacity of PEO-b-PCL with increasing PCL content for two hydrophobic cucurbitacin drugs. Biomacromolecules.

[67] Ma L., Deng L., Chen J. (2014). Applications of Poly(Ethylene Oxide) in Controlled Release Tablet Systems: A Review. Drug Dev. Ind. Pharm..

[68] Raeisi M., Tajik H., Yarahmadi A., Sanginabadi S. (2015). Antimicrobial Effect of Cinnamon Essential Oil Against Escherichia Coli and Staphylococcus Aureus. Health Scope.

